# The association between Life’s Crucial 9 (LC9) and low muscle mass (LMM): The mediating role of Dietary Inflammatory Index (DII)

**DOI:** 10.1097/MD.0000000000048405

**Published:** 2026-04-24

**Authors:** Yuliang Fang, Shaoqun Huang, Shuxian Xie, Hongyang Gong

**Affiliations:** a Department of Oncology Surgery, Fuzhou Hospital of Traditional Chinese Medicine Affiliated to Fujian University of Traditional Chinese Medicine, Fuzhou, Fujian Province, China; b Department of Physiology, College of Medicine, Chosun University, Gwangju, Republic of Korea.

**Keywords:** Dietary Inflammatory Index, Life’s Crucial 9, low muscle mass, mediation analysis, NHANES

## Abstract

Some studies suggest a potential link between cardiovascular health, inflammation, and low muscle mass (LMM). However, the mechanisms through which inflammation influences the relationship between cardiovascular health and LMM remain unclear. Life’s Crucial 9 (LC9) is a recently proposed method for assessing cardiovascular health. In addition, the Dietary Inflammatory Index (DII) is a novel, quantifiable measure of dietary inflammatory potential. This study investigates the relationship between LC9 and LMM and evaluates whether DII moderates this relationship. Subgroup analysis, restricted cubic splines, and multivariable logistic regression were employed to explore the relationship between LC9 and LMM. In addition, mediation analysis was conducted to investigate the potential role of DII in moderating the relationship between LC9 and LMM. Data were obtained from the National Health and Nutrition Examination Survey conducted between 2011 and 2018. A total of 7815 participants were included in this study, with 653 reporting LMM events. After adjusting for all variables using multivariable logistic regression, each 10-unit increase in LC9 was associated with a 53% reduction in LMM prevalence (odds ratio = 0.47, 95% confidence interval = 0.39–0.55), while each 1-unit increase in DII was associated with a 25% increase in LMM prevalence (odds ratio = 1.25, 95% confidence interval = 1.16–1.35). Consistent results were obtained when LC9 and DII were categorized into tertiles, with a *P* for trend <.001. Restricted cubic splines analysis revealed a linear negative association between LC9 and LMM prevalence. Mediation analysis indicated that 11.10% of the relationship between LC9 and LMM was mediated by DII (*P* = .002). This study found a significant negative association between LC9 and LMM, with DII partially mediating this relationship. These findings highlight the potential link between cardiovascular health and LMM, underscoring the importance of an anti-inflammatory diet in reducing the prevalence of LMM.

## 1. Introduction

Low muscle mass (LMM) is a common age-related degenerative condition characterized by a progressive decline in skeletal muscle mass and function.^[1]^ Due to different classifications and thresholds across various guidelines, the estimated prevalence of LMM ranges from 9.9% to 40.4%.^[2]^ With the global aging population and increasing life expectancy, it is currently estimated that 50 million people worldwide suffer from LMM, and this number is projected to exceed 500 million by 2050.^[3]^ LMM significantly impacts physical function and quality of life and is closely associated with higher disability rates and mortality risk.^[4]^ In addition, it places a heavy economic burden on healthcare systems. In the United States, the annual direct medical costs associated with LMM reach $18.5 billion (nearly 1.5% of total healthcare expenditures).^[5]^ This figure highlights the economic burden of LMM on public health systems and its impact on individual patients and societal health. Therefore, the prevention and management of LMM is not only a critical concern in healthcare but also an important socioeconomic issue.

Previous studies have established a strong association between LMM and various cardiovascular health factors, including hypertension, diabetes, and dyslipidemia.^[6]^ Research has also confirmed that LMM is significantly linked to an increased risk of cardiovascular events and mortality.^[7]^ In 2010, the American Heart Association (AHA) introduced “Life’s Simple 7,” which was later expanded to “Life’s Essential 8” with the inclusion of sleep health. More recently, the concept of “Life’s Crucial 9” was proposed, further emphasizing the importance of mental health in cardiovascular disease prevention.^[8]^ This framework provides a more comprehensive perspective on cardiovascular health assessment, incorporating not only traditional physiological markers but also lifestyle and mental health factors. However, despite the associations between individual components of Life’s Crucial 9 (LC9) and muscle health, the overall impact of LC9 on LMM remains insufficiently studied. This represents a critical knowledge gap in our understanding of the integration between muscle mass maintenance and cardiovascular health.

Inflammation plays a key role in the pathogenesis of LMM, as chronic low-grade inflammation leads to muscle protein breakdown and impaired muscle synthesis, negatively impacting muscle health.^[9]^ The Dietary Inflammatory Index (DII) provides a standardized method for assessing the inflammatory potential of an individual’s diet.^[10]^ While previous studies have examined the direct association between dietary patterns and muscle mass,^[11]^ the potential mediating role of DII in the relationship between LC9 and LMM remains unclear. The National Health and Nutrition Examination Survey (NHANES) 2011 to 2018 database offers a unique opportunity to explore this relationship, as it includes comprehensive dietary, clinical, and anthropometric data from a nationally representative sample. This study may provide new insights into the complex interactions between cardiovascular health factors, dietary inflammation, and muscle mass maintenance.

## 2. Methods

### 2.1. Study participants

The NHANES, conducted by the National Center for Health Statistics of the Centers for Disease Control and Prevention, is a nationwide health and nutrition survey program that began in the 1960s and became a continuous survey in 1999. NHANES is conducted biennially and collects data through questionnaires, physical exams, and laboratory tests, covering a wide range of areas, including demographics, nutrition intake, physical examinations, and laboratory data. The survey aims to monitor health trends and lifestyle changes in the U.S. population, providing data to support public health policy development. Written informed consent from each participant is necessary. The National Center for Health Statistics’ Research Ethics Review Board examines and approves the NHANES study to guarantee ethical compliance and scientific integrity.

This study is a cross-sectional study and employed a rigorous sample selection process to ensure data integrity and the reliability of the findings. Based on NHANES data from the 2011 to 2018 cycles, the initial sample included 39,156 participants. The selection process involved 3 major steps: first, participants under 20 years old and pregnant women were excluded (N = 16,539), leaving 22,617 nonpregnant adults; second, participants with incomplete LC9 and DII data were excluded (N = 8128), reducing the sample to 14,489 individuals; finally, individuals with incomplete data on LMM were excluded (N = 6674), resulting in a final study sample of 7815 (Fig. S1, Supplemental Digital Content, https://links.lww.com/MD/R714). These stringent selection steps not only ensured the representativeness of the sample but also provided a solid foundation for the scientific validity and credibility of the study results.

### 2.2. LMM assessment

Appendicular lean mass (ALM) is a key physiological indicator for assessing muscle mass, specifically referring to the nonfat tissue mass of the upper and lower limbs, primarily composed of skeletal muscle. In this study, ALM data were obtained through whole-body scans using dual-energy X-ray (Hologic, Inc., Bedford) absorptiometry from the NHANES program. The specific calculation method involves summing the lean body mass measurements of the right upper limb, left upper limb, right lower limb, and left lower limb. To enhance the accuracy of muscle status assessment, this study used the ratio of ALM to Body Mass Index (BMI; ALM/BMI) as the evaluation metric. This metric’s scientific value lies in its consideration of both absolute muscle mass and individual body composition differences, providing a more clinically meaningful muscle status assessment. According to the diagnostic criteria set by the Foundation for the National Institutes of Health,^[12,13]^ LMM is defined as follows: for men, ALM/BMI < 0.789; for women, ALM/BMI < 0.512. This ALM/BMI-based evaluation method integrates both muscle mass and body composition characteristics, offering a more objective and accurate basis for muscle status assessment in clinical practice and research.

### 2.3. LC9

LC9 is a comprehensive health assessment system,^[8]^ which includes 4 health behavior indicators (diet quality, physical activity, tobacco use, and sleep quality) and 5 health status indicators (weight status, blood lipid levels, blood glucose levels, blood pressure levels, and mental health status). Using the NHANES database, we quantified the LC9 indicators for each study participant, following previous research protocols^[14]^ (detailed scoring criteria are provided in Table S1, Supplemental Digital Content, https://links.lww.com/MD/R713). Each indicator is scored on a scale from 0 to 100, and the overall LC9 score is obtained by calculating the arithmetic average of the 9 individual indicators, creating a comprehensive quantitative metric that reflects an individual’s overall health status. Specifically, diet quality was assessed using the Healthy Eating Index-2015^[15]^ (scoring components and standards are detailed in Table S2, Supplemental Digital Content, https://links.lww.com/MD/R713). Data on sleep quality (self-reported average hours of sleep per night), tobacco use (self-reported use of cigarettes or inhaled nicotine-delivery system), physical activity level (self-reported minutes of moderate or vigorous physical activity per week), and mental health status (9-item depression screening instrument Patient Health Questionnaire-9 were obtained from NHANES standardized questionnaire surveys. Objective measures of weight status (BMI), blood pressure levels, blood glucose levels, and blood lipid levels (serum cholesterol levels) were taken by certified healthcare professionals according to NHANES standard operating procedures. All data are available for verification in the official NHANES database (https://wwwn.cdc.gov/nchs/nhanes/).

### 2.4. Definition of DII

The DII is a scientifically quantifiable tool used to assess the inflammatory potential of diet, developed by a research team at the University of South Carolina.^[15]^ The index quantifies the pro-inflammatory or anti-inflammatory potential of a diet by systematically evaluating the impact of dietary components on specific inflammatory biomarkers. A DII score ≥0 indicates a pro-inflammatory diet, while a score <0 represents an anti-inflammatory diet. The higher the absolute value of the DII score, the stronger the pro-inflammatory or anti-inflammatory potential of the diet. The DII framework consists of 45 dietary parameters, and this study utilized 28 available parameters from the NHANES 24-hour dietary recall data (24HR), including carbohydrates, protein, total fat, cholesterol, saturated fatty acids, monounsaturated fatty acids, polyunsaturated fatty acids, n-3 fatty acids, n-6 fatty acids, vitamins A, D, E, B1, B2, B3, B6, B12, C, β-carotene, folate, iron, magnesium, selenium, zinc, dietary fiber, energy, alcohol, and caffeine. Notably, previous studies have shown that the DII score calculated using only these 28 parameters remains a reliable predictor,^[16]^ effectively assessing the inflammatory potential of a diet. The specific algorithm for calculating the DII score is provided in the supplementary materials.

### 2.5. Covariables

The covariates in this study included age, sex, education level, marital status, family poverty-income ratio (PIR), race, smoking, alcohol consumption, physical activity, hypertension, diabetes, and hyperlipidemia. Detailed information on these covariates can be found in Table S3, Supplemental Digital Content, https://links.lww.com/MD/R713.

### 2.6. Statistical analysis

To ensure the data represented the national population, all analyses were conducted using sampling weights. The weight variable used in our study was the 2-day dietary sample weight (WTDR2D), and the new weight for the 2011 to 2018 period was calculated as 1/4 × WTDR2D. Continuous variables are presented as mean ± standard deviation, and categorical variables are presented as frequencies (percentages). Comparisons of continuous variables were conducted using weighted *t* tests, while categorical variables were compared using weighted chi-square tests. Weighted logistic regression was used to explore the relationship between LC9 and LMM. Three logistic regression models were established: Model 1: no adjustment for potential confounders; Model 2: adjustment for covariates including age, sex, education level, marital status, PIR, and race; Model 3: further adjustment for smoking, alcohol consumption, physical activity, hypertension, diabetes, and hyperlipidemia in addition to Model 2. In Model 3, LC9 was treated as a continuous variable, and restricted cubic splines were used to illustrate the linear or nonlinear association between LC9 and LMM. Subsequently, subgroup analyses were performed based on Model 3 to explore potential variations in the association across subgroups.

Based on the premise that “LC9 is significantly associated with DII” and “DII is significantly associated with LMM,” a mediation analysis was conducted to examine whether the effect of LC9 on LMM is mediated by DII. The mediation effect was calculated using the “mediation” package in R software. Data processing was conducted using R statistical software (version 4.3.1). A two-sided *P*-value of <.05 was considered statistically significant.

## 3. Result

### 3.1. Baseline characteristics

This study included 7815 participants aged 20 years and older, representing approximately 96.41 million U.S. adults. The prevalence of LMM was 7%, corresponding to approximately 6.78 million individuals. In addition, the LC9 scores in the LMM group were lower than those in the non-LMM group, while the DII scores were higher in the LMM group compared to the non-LMM group. Significant differences between the LMM and non-LMM groups were observed in terms of age, race, education level, PIR, alcohol consumption, hypertension, diabetes, and hyperlipidemia (all *P* < .05). More detailed information can be found in Table 1.

**Table 1:** Baseline characteristics of all participants were stratified by low muscle mass, weighted.

Characteristic	Overall, N = 96,416,743 (100%)	Non-low muscle, N = 89,647,310 (93%)	Low muscle mass, N = 6,769,433 (7%)	*P* value
No. of participants in the sample	7815	7162	653	–
Age (%)				**<.001**
20–40	49,182,735 (51%)	46,600,827 (52%)	2,581,908 (38%)	
>40	47,234,008 (49%)	43,046,483 (48%)	4,187,525 (62%)	
Gender (%)				.185
Male	47,875,249 (50%)	44,263,117 (49%)	3,612,133 (53%)	
Female	48,541,494 (50%)	45,384,194 (51%)	3,157,300 (47%)	
Race (%)				**<.001**
Non-Hispanic White	61,108,367 (63%)	57,677,077 (64%)	3,431,290 (51%)	
Other	15,427,921 (16%)	14,004,893 (16%)	1,423,028 (21%)	
Non-Hispanic Black	10,452,183 (11%)	10,193,666 (11%)	258,518 (4%)	
Mexican American	9,428,272 (10%)	7,771,674 (9%)	1,656,598 (24%)	
Married/live with partner (%)				.725
No	35,930,755 (37%)	33,347,070 (37%)	2,583,685 (38%)	
Yes	60,485,989 (63%)	56,300,240 (63%)	4,185,748 (62%)	
Education level (%)				**<.001**
Below high school	10,872,688 (11%)	9,447,293 (11%)	1,425,395 (21%)	
High school or above	85,544,055 (89%)	80,200,018 (89%)	5,344,038 (79%)	
PIR (%)				**<.001**
Not poor	70,894,910 (78%)	66,508,159 (79%)	4,386,751 (70%)	
Poor	19,789,412 (22%)	17,867,364 (21%)	1,922,048 (30%)	
Smoking (%)				.222
Never	57,322,598 (59%)	53,251,573 (59%)	4,071,025 (60%)	
Former	19,263,582 (20%)	17,746,092 (20%)	1,517,490 (23%)	
Current	19,830,563 (21%)	18,649,645 (21%)	1,180,918 (17%)	
Drinking (%)				**<.001**
Former	7,851,674 (8%)	6,964,695 (8%)	886,979 (14%)	
Heavy	24,433,343 (26%)	22,771,206 (26%)	1,662,137 (26%)	
Mild	33,055,227 (35%)	31,252,388 (36%)	1,802,838 (28%)	
Moderate	19,391,658 (21%)	18,496,480 (21%)	895,178 (14%)	
Never	9,012,975 (10%)	7,828,467 (9%)	1,184,508 (18%)	
Physical activity (%)				.307
Inactive	11,510,676 (14%)	10,756,105 (14%)	754,571 (16%)	
Active	69,254,820 (86%)	65,390,660 (86%)	3,864,159 (84%)	
Hypertension (%)				**<.001**
No	70,578,057 (73%)	66,589,491 (74%)	3,988,566 (59%)	
Yes	25,838,687 (27%)	23,057,820 (26%)	2,780,867 (41%)	
Diabetes (%)				**<.001**
No	89,212,548 (93%)	83,694,364 (93%)	5,518,184 (82%)	
Yes	7,204,196 (7%)	5,952,946 (7%)	1,251,249 (18%)	
Hyperlipidemia (%)				**<.001**
No	35,908,269 (37%)	34,253,327 (38%)	1,654,942 (24%)	
Yes	60,508,474 (63%)	55,393,983 (62%)	5,114,491 (76%)	
LC9 (mean [SD])	73.05 (13.59)	73.73 (13.38)	63.98 (13.18)	**<.001**
LC9, tertile (%)				**<.001**
T1	32,723,670 (34%)	28,544,427 (32%)	4,179,244 (62%)	
T2	30,869,349 (32%)	28,963,597 (32%)	1,905,752 (28%)	
T3	32,823,724 (34%)	32,139,286 (36%)	684,438 (10%)	
DII (mean [SD])	1.21 (1.85)	1.17 (1.86)	1.70 (1.66)	**<.001**
DII, tertile (%)				**<.001**
T1	32,134,221 (33%)	30,591,357 (34%)	1,542,864 (23%)	
T2	32,168,656 (34%)	29,733,255 (33%)	2,435,400 (36%)	
T3	32,113,867 (33%)	29,322,698 (33%)	2,791,169 (41%)	

Mean (SD) for continuous variables: the *P* value was calculated by the weighted Students *t* test. Percentages (weighted N, %) for categorical variables: the *P* value was calculated by the weighted chi-square test.

DII = Dietary Inflammatory Index, LC9 = Life’s Crucial 9, PIR = poverty-income ratio, SD = standard deviation.

### 3.2. The association between LC9, DII, and LMM

**Figure 1.**
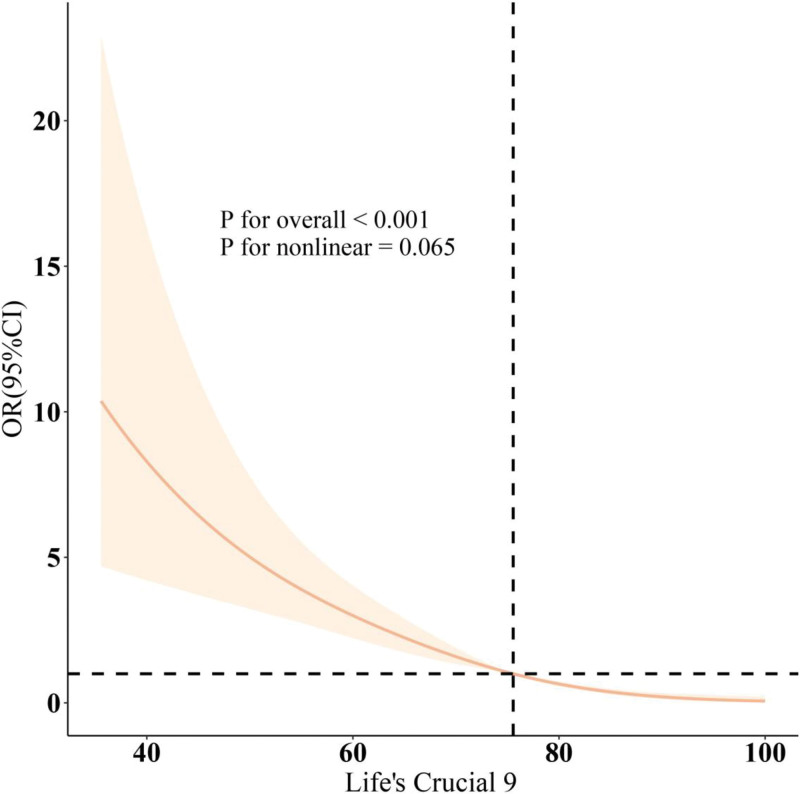
Dose–response relationships between LC9 and low muscle mass. OR (solid lines) and 95% confidence levels (shaded areas) were adjusted for age, gender, education level, marital, PIR, race, smoking, drinking, physical activity, hypertension, diabetes, and hyperlipidemia. CI = confidence interval, LC9 = Life’s Crucial 9, OR = odds ratio, PIR = poverty-income ratio.

As shown in Table 2, 3 different models were used to assess the association between LC9 scores and the prevalence of LMM. All models indicated a negative correlation between LC9 scores and LMM prevalence (*P* < .001). In Model 3, after adjusting for various covariates, every 10-point increase in LC9 score was associated with a 53% reduction in LMM prevalence (odds ratio [OR] = 0.47, 95% confidence interval [CI] = 0.39–0.55). Furthermore, when LC9 was converted into quartiles, the group with the highest LC9 scores (T3) had an 88% lower prevalence of LMM compared to the group with the lowest LC9 scores (T1; OR = 0.12, 95% CI = 0.07–0.20). In addition, the relationship between DII and LMM was assessed, and the results showed a positive association between DII and LMM prevalence across all 3 models (all *P* < .05). As DII scores increased, the prevalence of LMM also increased, with statistically significant differences observed (*P* < .05). As illustrated in Figure 1, after adjusting for related variables, the negative correlation between LC9 scores and LMM prevalence was further confirmed (overall *P* < .001; nonlinear *P* = .065).

**Table 2:** Association between LC9, DII, and low muscle mass, NHANES 2011 to 2018.

Characteristics	Model 1 (OR [95% CI])	*P* value	Model 2 (OR [95% CI])	*P* value	Model 3 (OR [95% CI])	*P* value
LC9 – low muscle mass						
Continuous (per 10 scores)	0.61 (0.58–0.65)	<.001	0.63 (0.60–0.68)	<.001	0.47 (0.39–0.55)	<.001
Tertile						
T1	1 (ref.)		1 (ref.)		1 (ref.)	
T2	0.45 (0.37–0.55)	<.001	0.47 (0.38–0.59)	<.001	0.37 (0.26–0.53)	<.001
T3	0.15 (0.11–0.20)	<.001	0.17 (0.12–0.23)	<.001	0.12 (0.07–0.20)	<.001
*P* for trend	<.001		<.001		<.001	
DII – low muscle mass						
Continuous	1.18 (1.10–1.25)	<.001	1.21 (1.13–1.29)	<.001	1.25 (1.16–1.35)	<.001
Tertile						
T1	1 (ref.)		1 (ref.)		1 (ref.)	
T2	1.62 (1.21–2.19)	.002	1.73 (1.26–2.36)	<.001	1.79 (1.22–2.61)	.004
T3	1.89 (1.40–2.54)	<.001	2.12 (1.55–2.90)	<.001	2.50 (1.73–3.60)	<.001
*P* for trend	<.001		<.001		<.001	

Model 1: no covariates were adjusted. Model 2: age, gender, education level, marital, PIR, and race were adjusted. Model 3: age, gender, education level, marital, PIR, race, smoking, drinking, physical activity, hypertension, diabetes, and hyperlipidemia were adjusted.

CI = confidence interval, DII = Dietary Inflammatory Index, LC9 = Life’s Crucial 9, NHANES = National Health and Nutrition Examination Survey, OR = odds ratio, PIR = poverty-income ratio.

### 3.3. Subgroup analysis

**Figure 2.**
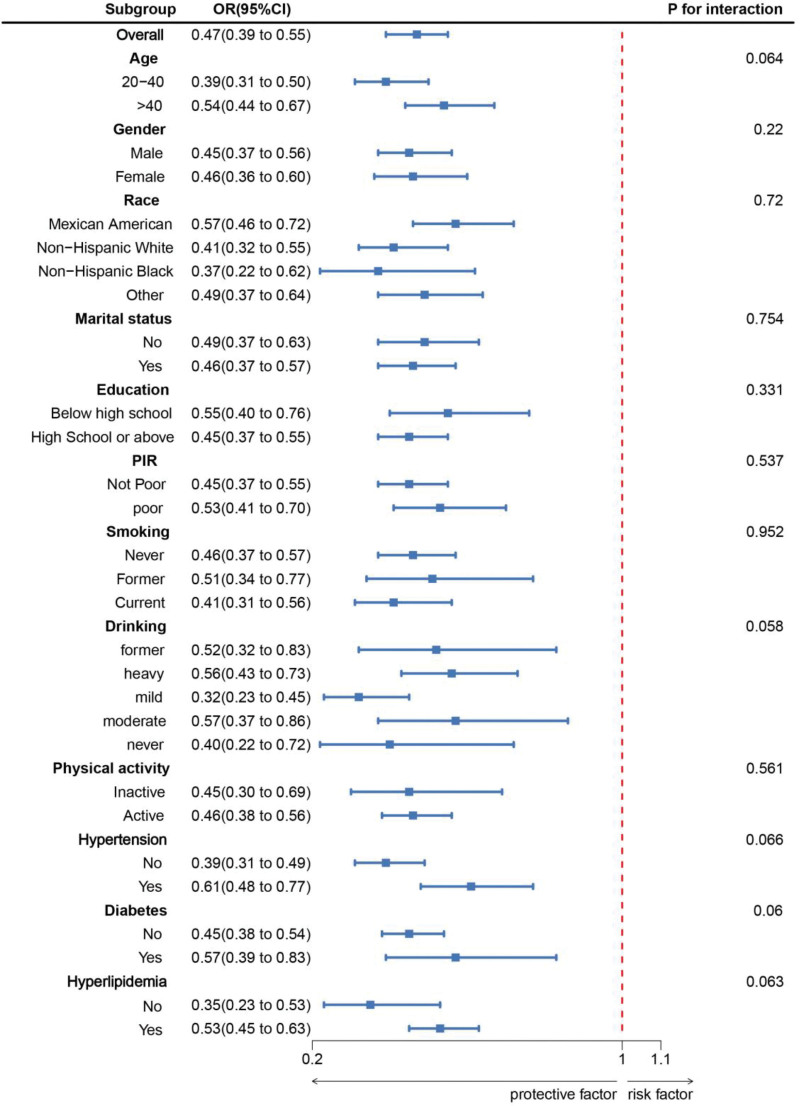
Subgroup analysis between LC9 and low muscle mass. ORs were calculated as per 10-unit increase in LC9. Analyses were adjusted for age, gender, education level, marital, PIR, race, smoking, drinking, physical activity, hypertension, diabetes, and hyperlipidemia. CI = confidence interval, LC9 = Life’s Crucial 9, OR = odds ratio, PIR = poverty-income ratio.

As shown in Figure 2, subgroup analyses were conducted based on age, sex, race, marital status, education level, PIR, smoking, alcohol consumption, physical activity, hypertension, diabetes, and hyperlipidemia. The results revealed a consistent negative correlation between LC9 scores and LMM across all subgroups. After adjusting for various confounders, no significant interactions were observed, indicating the stability of the association between LC9 and LMM.

### 3.4. Mediation effect

**Figure 3.**
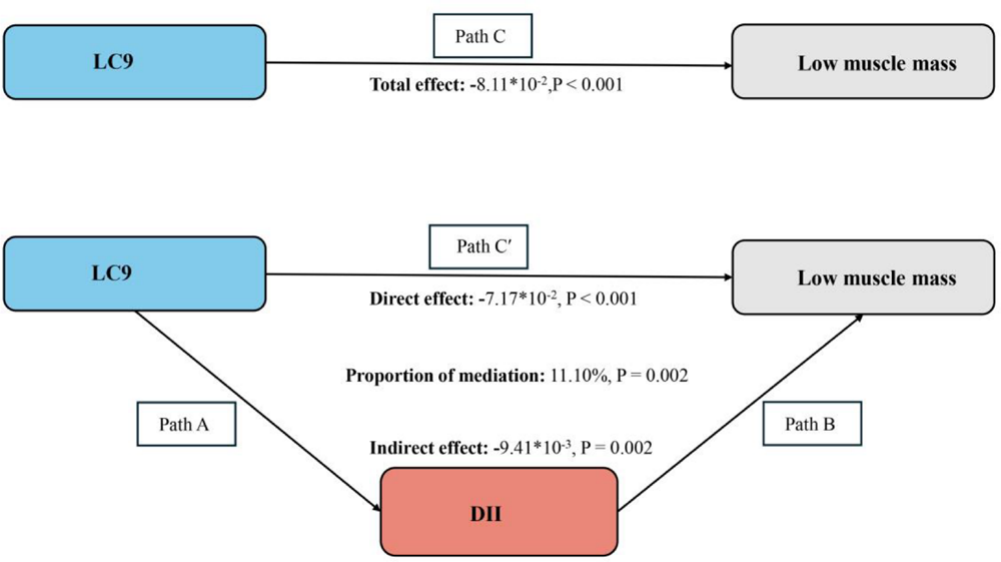
Schematic diagram of the mediation effect analysis. Path C indicates the total effect; path C′ indicates the direct effect. The indirect effect is estimated as the multiplication of paths A and B (path A × B). The mediated proportion is calculated as indirect effect/(indirect effect + direct effect) × 100%. Analyses were adjusted for age, gender, education level, marital, PIR, race, smoking, drinking, physical activity, hypertension, diabetes, and hyperlipidemia. DII = Dietary Inflammatory Index, LC9 = Life’s Crucial 9, PIR = poverty-income ratio.

The mediation model and pathway are illustrated in Figure 3, where LC9, LMM, and DII serve as the independent variable, dependent variable, and mediator, respectively. As shown in Table 3, after adjusting for other covariates, a significant correlation was found between LC9 and DII (β = −0.67, 95% CI = −0.74 to −0.60). After adjusting for all covariates, DII mediated 11.10% of the association between LC9 and LMM (indirect effect = −9.41 × 10^−3^, *P* = .002; direct effect = −7.17 × 10^−2^, *P* < .001), with the mediation ratio calculated as indirect effect/(indirect effect + direct effect) × 100%, *P* = .002 (Fig. 3). Thus, DII can be considered a mediator in the association between LC9 and LMM.

**Table 3:** Multivariate linear regression of LC9 and DII.

	β	95% CI	*P* value
LC9 – DII	−0.67	−0.74 to −0.60	<.001

Adjusted for age, gender, education level, marital, PIR, race, smoking, drinking, physical activity, hypertension, diabetes, and hyperlipidemia.

CI = confidence interval, DII = Dietary Inflammatory Index, LC9 = Life’s Crucial 9.

## 4. Discussion

In this study, we found a significant negative correlation between a healthy lifestyle (quantified by the LC9 score) and the risk of LMM. More importantly, we are the first to demonstrate that the DII plays a significant mediating role in the relationship between LC9 and LMM. Our findings suggest that higher LC9 scores may reduce the incidence of LMM by lowering dietary inflammation. These results underscore the potential impact of comprehensive lifestyle management on maintaining muscle mass and highlight the importance of monitoring and reducing the risk of LMM through the LC9 score.

In recent years, there has been extensive and in-depth research on the relationship between cardiovascular health and muscle health. Numerous epidemiological and clinical studies have confirmed that cardiovascular diseases are associated with LMM. A study involving individuals aged 80 and older showed a negative correlation between muscle mass and coronary artery calcification scores, with an OR for muscle mass of 2.54 (95% CI = 1.06–6.06).^[17]^ A study in the UK followed 4252 older men and found that sarcopenia and abdominal obesity were strongly associated with all-cause mortality and cardiovascular disease mortality, with higher all-cause mortality observed in patients with sarcopenic obesity.^[18]^ LC9 is an innovative health assessment system that integrates mental health into the cardiovascular health indicators of Life’s Essential 8 proposed by the AHA. A prospective cohort study indicated that LC9 scores were associated with both cardiovascular and all-cause mortality.^[14]^ Our study is the first to apply LC9, a comprehensive lifestyle score system, to muscle mass research, systematically assessing the cumulative effects of multidimensional lifestyle factors on muscle mass. We also innovatively explore the mediating role of inflammation pathways in this relationship. This research design not only expands the application of LC9 but also provides new perspectives on the cardiovascular-inflammation-muscle mass mechanistic relationship.

Microcirculatory dysfunction caused by cardiovascular diseases is one of the key mechanisms affecting muscle health. Under normal physiological conditions, endothelial cells regulate vascular tone and hemodynamics by producing vasodilatory substances such as nitric oxide, prostacyclin (PGI2), and endothelium-derived hyperpolarizing factor.^[19]^ However, in cardiovascular disease states, endothelial cell dysfunction leads to significant alterations in the production and release of these vasoregulatory substances. Notably, a decrease in the activity of endothelial nitric oxide synthase results in reduced bioavailability of nitric oxide, which not only impairs vascular relaxation but also exacerbates inflammation and oxidative stress.^[20]^ Microcirculatory dysfunction is also manifested in vascular remodeling. Studies have shown a significant reduction in capillary density in muscle tissues of cardiovascular disease patients, which is associated with the inhibition of vascular endothelial growth factor signaling pathways.^[21]^ The reduced vascular density directly impacts oxygen supply and nutrient delivery to muscle tissues, leading to chronic hypoxia in muscle cells, which hinders protein synthesis and energy metabolism. In addition, microcirculatory dysfunction affects the clearance of metabolic waste from muscle tissues.^[22]^ Slower blood flow and increased vascular permeability cause the accumulation of metabolic products in the local environment, leading to acidosis and ionic imbalance. These changes further impair muscle cell function, creating a vicious cycle.

Insulin-like growth factor-1 (IGF-1) is a multifunctional cell proliferation regulator widely distributed across various tissues. It plays a crucial role in cellular differentiation, proliferation, and individual growth and development.^[23]^ Research has demonstrated that IGF-1 is involved in cardiovascular diseases^[24]^ and bone tissue.^[25]^ IGF-1 promotes the synthesis of muscle fibers in skeletal muscle cells and guides the growth and differentiation of muscle satellite cells. A decrease in IGF-1 levels is associated with reduced muscle mass and strength. Hambrecht et al^[26]^ found that in heart failure patients, the mRNA expression of IGF-1 receptors in muscle decreased by 52%, which may contribute to early muscle loss. Cardiovascular diseases often involve sustained activation of the sympathetic nervous system, leading to elevated levels of norepinephrine and epinephrine.^[27]^ The continuous activation of the sympathetic nervous system and the renin-angiotensin system can lead to muscle mass loss. In animal studies, mice injected with angiotensin I showed significant reductions in body weight and skeletal muscle mass, and researchers observed a decline in IGF-1 levels, which are involved in promoting skeletal muscle synthesis.^[28]^ Recent studies have also shown that insulin receptor substrate 1, as a downstream mediator of IGF-1, when degraded, leads to a decline in protein synthesis in skeletal muscle cells. In addition, IGF-1 can inhibit protein degradation and promote muscle growth.^[29]^ Under conditions of muscle atrophy, the IGF-1 signaling pathway also plays a significant role in muscle wasting, for example, by transferring activated Akt into muscle fibers via electroporation, which can suppress denervation-induced muscle atrophy.^[30]^

In recent years, there has been increasing attention in the medical field on the impact of mental health on the cardiovascular system. Building on the AHA’s “Life’s Essential 8,” mental health has been included in the framework of “Life’s Crucial 9,” emphasizing its importance in overall health. Research has shown that prolonged psychological stress, depression, and anxiety are significantly associated with the incidence of cardiovascular diseases.^[31]^ Moreover, mental health factors such as depression^[32]^ and anxiety^[33]^ have been proven to be strongly linked to muscle health. When individuals are under chronic stress, the hypothalamic-pituitary-adrenal axis and other stress response systems are activated. This activation leads to the increased secretion of various inflammatory factors, particularly pro-inflammatory cytokines such as interleukin-6 (IL-6) and tumor necrosis factor-α (TNF-α).^[34]^ This systemic inflammatory response can adversely affect multiple organ systems, including muscle tissue. In addition, psychological issues such as depression and anxiety often result in decreased appetite, leading to inadequate nutritional intake. A deficiency in nutrients like protein directly impacts muscle maintenance and growth. Furthermore, psychological stress may also contribute to overeating or disordered eating, preventing the body from receiving a balanced supply of nutrients, which in turn affects muscle health.

Our study is the first to explore the mediating role of the DII in the relationship between LC9 and LMM, offering new insights into how lifestyle factors may influence muscle health through inflammatory pathways. Inflammation, as a critical immune defense mechanism, can lead to tissue damage when it becomes excessive or chronic. Imbalances in inflammatory mediators play a key role in the reduction of muscle mass. The excessive and prolonged release of inflammatory mediators such as TNF-α, IL-4, IL-6, IL-10, IL-15, C-reactive protein, and leptin can cause cellular damage and muscle loss, contributing to the decrease in muscle mass and strength observed in older adults.^[9,35,36]^ In addition, TNF-α activates the NF-κB signaling pathway via TNF receptor-associated factor 6, inducing the expression of muscle atrophy-related genes *MuRF-1* and *MAFbx*, which promote skeletal muscle fiber degradation through the ubiquitin-proteasome system.^[37]^ Based on these findings, managing chronic inflammation through lifestyle modifications may serve as an effective strategy for preventing and treating LMM. This research not only enhances our understanding of the factors influencing muscle health but also provides novel intervention strategies for managing the health of elderly populations.

The findings of this study hold significant public health and clinical practice implications. First, by identifying the mediating role of DII in the relationship between LC9 and LMM, we provide a new perspective for the prevention and intervention of LMM. This suggests that, in addition to traditional lifestyle management, controlling diet-related inflammation may serve as an effective intervention target. Healthcare professionals should consider the anti-inflammatory properties of diet as a key factor when developing prevention strategies. Second, this study underscores the importance of integrated management. LC9, as a comprehensive cardiovascular health indicator, is associated with muscle health, emphasizing the need for an integrated approach in clinical practice. Muscle mass management should be incorporated into the framework of cardiovascular health management. This holistic management approach benefits cardiovascular health and helps maintain muscle mass by improving inflammatory states. Third, the findings provide specific guidance for nutrition interventions. Adjusting dietary patterns to increase the intake of anti-inflammatory nutrients (such as fruits, vegetables, whole grains, and fish) while reducing pro-inflammatory foods (like refined carbohydrates and saturated fats) could help prevent and improve LMM. These recommendations are easy to understand and implement, offering strong practical feasibility. Lastly, the results support the use of multi-level intervention strategies in public health initiatives. At the community level, health education can raise awareness of the relationship between dietary inflammation and muscle health; at healthcare institutions, DII assessment can be integrated into routine health checks to provide targeted nutritional guidance for high-risk groups; and at the policy level, promoting anti-inflammatory dietary patterns can be considered a part of healthy aging strategies. Implementing these measures is expected to yield positive health benefits at the population level.

This study offers several notable strengths. First, it is based on the nationally representative NHANES 2011 to 2018 database, a large-scale dataset that enhances statistical power, reduces random error and ensures robust external validity due to the demographic diversity of the sample. In addition, NHANES’s stringent quality control measures and standardized protocols guarantee the reliability and accuracy of the data. Second, this study is the first to explore the mediating role of the DII in the relationship between LC9 and LMM, representing a significant innovation. This innovation lies not only in the research perspective but, more importantly, in revealing the potential mechanisms by which lifestyle factors influence muscle health, offering a new theoretical framework for the field. By incorporating DII as a mediating variable, this study bridges the gap between LC9 and LMM, providing a deeper understanding of their relationship. Third, the study employs rigorous statistical methodology. In the mediation analysis, we examine the total effect, direct effect, and indirect effect to ensure the robustness of the results. In addition, we carefully control for potential confounders in model adjustments, which enhances the internal validity of the findings. Lastly, this study simultaneously considers both the overall LC9 score and its individual components in relation to LMM, providing a more comprehensive and nuanced analysis. This multi-layered approach allows us to evaluate the impact of overall lifestyle factors while identifying specific elements that most significantly affect muscle health, offering more targeted guidance for clinical practice. The strength of this analytical strategy is its ability to provide valuable insights at both the macro and micro levels.

This study has several limitations that should be considered. First, due to the cross-sectional design, it is not possible to establish causality or the temporal sequence between LC9, DII, and LMM; the observed associations may be influenced by reverse causality. Second, although multiple confounders were adjusted for in the analysis, there may still be unmeasured or unidentified confounders that could affect the results. In addition, some variables in the NHANES database, such as dietary intake and physical activity levels, rely on self-reports, which may introduce recall bias or reporting bias. Furthermore, the DII score is based solely on available dietary data, which may not fully capture an individual’s actual inflammatory dietary state. In addition, assessment of dietary data from 24-hour recall may not be ideal method when assessing the relation of diet and cardiovascular outcome and long term diet would affect such chronic outcomes rather than a single day diet which may not faithfully reflect true diet of the person. While the NHANES data are nationally representative, the results are primarily applicable to the U.S. population, and caution should be taken when generalizing the findings to other racial or demographic groups. Finally, the study lacked certain important biomarkers that could influence the results, such as inflammatory cytokine levels, which limits a deeper exploration of the mechanisms underlying the mediating effects.

## 5. Conclusion

In conclusion, this study, based on the NHANES 2011 to 2018 database, is the first to explore the mediating role of DII in the relationship between LC9 and LMM. The study found that LC9 score is significantly associated with LMM, and this relationship is partially mediated through DII, suggesting that diet-related inflammation plays a crucial role in linking lifestyle factors to muscle health. This finding not only deepens our understanding of the mechanisms through which lifestyle impacts muscle mass but also provides a novel intervention target for the prevention and improvement of LMM. The innovation of this study lies in integrating 3 important health indicators – LC9, DII, and muscle mass – into a unified theoretical framework, revealing the intrinsic connections among them. These findings hold significant public health implications, highlighting the importance of focusing on lifestyle improvement and diet’s anti-inflammatory properties when preventing LMM. Future prevention strategies could incorporate anti-inflammatory diets as a key component, likely enhancing intervention effectiveness and improving overall population health.

## Acknowledgments

We sincerely appreciate the NHANES database for all the data.

## Author contributions

**Conceptualization:** Yuliang Fang, Shaoqun Huang, Shuxian Xie, Hongyang Gong.

**Data curation:** Yuliang Fang, Shaoqun Huang, Shuxian Xie, Hongyang Gong.

**Resources:** Yuliang Fang, Hongyang Gong.

**Software:** Yuliang Fang, Hongyang Gong.

**Funding acquisition:** Shaoqun Huang.

**Investigation:** Shaoqun Huang, Hongyang Gong.

**Methodology:** Shaoqun Huang, Hongyang Gong.

**Supervision:** Shaoqun Huang.

**Visualization:** Shaoqun Huang, Hongyang Gong.

**Writing – review & editing:** Shaoqun Huang.

**Formal analysis:** Shuxian Xie, Hongyang Gong.

**Validation:** Hongyang Gong.

**Writing – original draft:** Hongyang Gong.

## Supplementary Material
