# Supplementary material for: The association between Life’s Crucial 9 (LC9) and low muscle mass (LMM): The mediating role of Dietary Inflammatory Index (DII)
Source: Medicine (Baltimore). 2026 Apr 24;105(17):e48405. doi: 10.1097/MD.0000000000048405 (PMC13124353; doi:10.1097/MD.0000000000048405)
Supplement: Supplementary file 1 [file medi-105-e48405-s001.pdf]

Supplementary Material

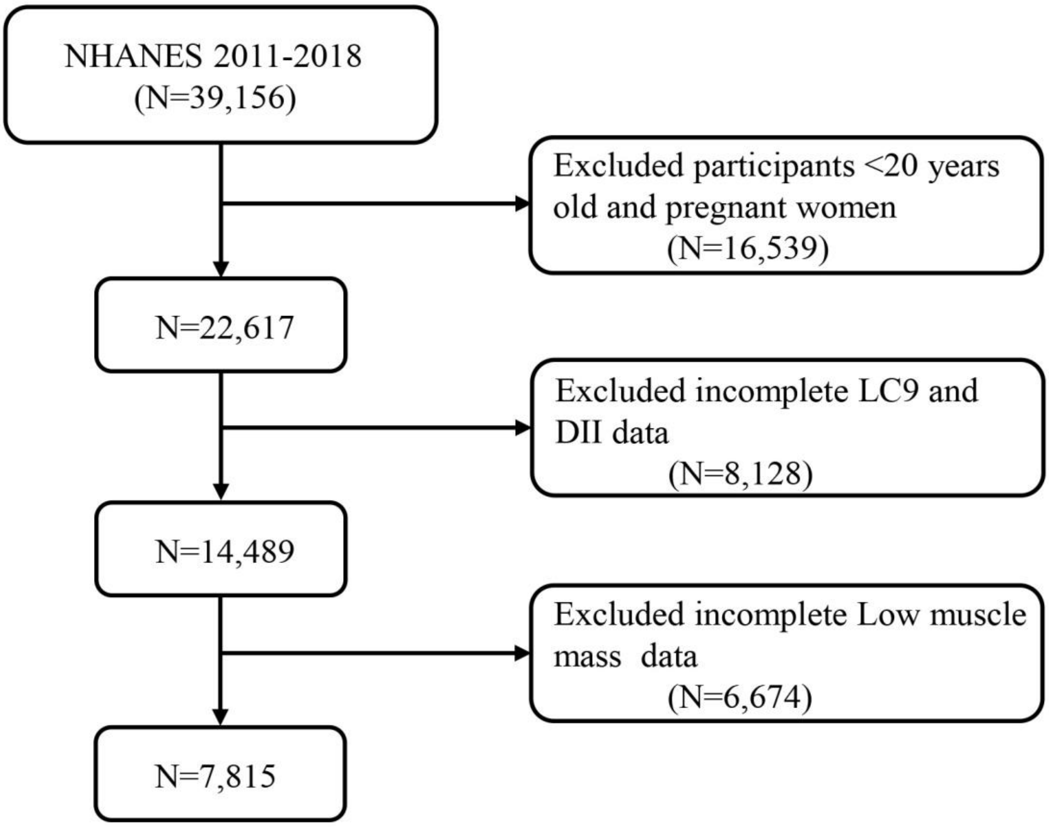

Figure S1. A flow diagram of eligible participant selection in the National Health and Nutrition Examination Survey.

Abbreviation: LC9, Life's Crucial 9; DII, Dietary Inflammatory Index.
