# Supplementary material for: The association between Life’s Crucial 9 (LC9) and low muscle mass (LMM): The mediating role of Dietary Inflammatory Index (DII)
Source: Medicine (Baltimore). 2026 Apr 24;105(17):e48405. doi: 10.1097/MD.0000000000048405 (PMC13124353; doi:10.1097/MD.0000000000048405)
Supplement: Supplementary file 2 [file medi-105-e48405-s002.pdf]

Supplementary Material

Calculation of the dietary inflammatory index(DII)[1].

Calculation of the DII is based on dietary intake data that are then linked to the regionally representative world database that provided a robust estimate of a mean and standard deviation for each parameter. These then become the multipliers to express an individual's exposure relative to the 'standard global mean' as a Z-score. This is achieved by subtracting the 'standard mean' from the amount reported and dividing this value by its standard deviation. To minimize the effect of 'right skewing', this value is converted to a percentile score. To achieve a symmetrical distribution with values centred on 0 (null) and bounded between −1 (maximally anti-inflammatory) and +1 (maximally pro-inflammatory), each percentile score is doubled and then '1' is subtracted.

The centred percentile value for each food parameter is then multiplied by its respective 'overall food parameter-specific inflammatory effect score' to obtain the 'food parameter-specific DII score'. Finally, all of the 'food parameter-specific DII scores' are summed to create the 'overall DII score' for an individual.

The 28 nutrients in this study were alcohol, β-carotene, caffeine, carbohydrate, cholesterol, energy, total fat, fiber, folic acid, iron, magnesium, monounsaturated fatty acids, polyunsaturated fatty acids, n-3 fatty acids, n-6 fatty acids, protein, saturated fat, selenium, zinc, vitamin A, B1, B2, B3, B6, B12, C, D, and E[2].

1. Shivappa N, Steck SE, Hurley TG, Hussey JR, Hébert JR. Designing and developing a literature-derived, population-based dietary inflammatory index. Public Health Nutrition. 2014;17:1689–96.

2. Meng X, Sha W, Lou X, Chen J. The relationship between dietary inflammatory index and osteoporosis among chronic kidney disease population. Sci Rep. 2023;13:22867.

Table S1. Definition and scoring approach for the Life's Crucial 9 score.

Domain	CVH Metric	Measurement	Quantification and Scoring of CVH Metric
Psychological Health	Depressive symptoms	Depression Screener Questionnaire (DPQ)	**Metric:** Nine-item depression screening instrument PHQ-9. Each symptom item in PHQ-9 is scored on a 4-point scale, from 0 ('not at all') to 3 ('nearly every day'), resulting in a total score of 0 to 27 points.<br><br>**Scoring:**<br>Points Level<br>100 the score of 0 to 4 points<br>70 the score of 5 to 9 points<br>30 the score of 10 to 14 points<br>0 the score of 15 to 27 points
Health Behaviors	Diet	Healthy Eating	Quantiles of DASH-style diet adherence

		Index-2015 diet score percentile	**Scoring (Population):**<br><u>Points</u> <u>Quantile</u><br>100 ≥95th percentile (top/ideal diet)<br>80 75th – 94th percentile<br>50 50th – 74th percentile<br>25 25th – 49th percentile<br>0 1st – 24th percentile (bottom/least ideal quartile)
	Physical activity	Self-reported minutes of moderate or vigorous physical activity per week	**Metric:** Minutes of moderate (or greater) intensity activity per week<br><br>**Scoring:**<br><u>Points</u> <u>Minutes</u><br>100 ≥150<br>90 120 – 149<br>80 90 – 119<br>60 60 – 89<br>40 30 – 59<br>20 1 – 29<br>0 0
	Nicotine exposure	Self-reported use of cigarettes or inhaled nicotine-delivery system	**Metric:** Combustible tobacco use and inhaled NDS use; or secondhand smoke exposure<br><br>**Scoring:**<br><u>Points</u> <u>Status</u><br>100 Never smoker<br>75 Former smoker, quit ≥5 yrs<br>50 Former smoker, quit 1 - <5 yrs<br>25 Former smoker, quit <1 year, or currently using inhaled NDS<br>0 Current smoker<br><br>Subtract 20 points (unless the score is 0) for living with an active indoor smoker in the home
	Sleep health	Self-reported average hours of sleep per night	**Metric:** Average hours of sleep per night<br><br>**Scoring:**<br><u>Points</u> <u>Level</u><br>100 7 – <9<br>90 9 – <10<br>70 6 – <7<br>40 5 – <6 or ≥10

			20 4 – <5<br>0 <4
Health Factors	Body mass index	Body weight (kg) divided by height squared (m^2)	**Metric:** Body mass index (kg/m^2)<br><br>**Scoring:**<br><u>Points Level</u><br><br>100 <25.0<br>70 25.0 – 29.9<br>30 30.0 – 34.9<br>15 35.0 – 39.9<br>0 ≥40.0
	Blood lipids	Plasma total and HDL-cholesterol with the calculation of non-HDL-cholesterol	**Metric:** Non-HDL-cholesterol (mg/dL)<br><br>**Scoring:**<br><u>Points Level</u><br>100 <130<br>60 130 – 159<br>40 160 – 189<br>20 190 – 219<br>0 ≥220<br><br>If the drug-treated level, subtract 20 points
	Blood glucose	Fasting blood glucose or casual hemoglobin A1c	**Metric:** Fasting blood glucose (mg/dL) or Hemoglobin A1c (%)<br><br>**Scoring:**<br><u>Points Level</u><br>100 No history of diabetes and FBG <100 (or HbA1c < 5.7)<br>60 No diabetes and FBG 100 – 125 (or HbA1c 5.7-6.4) (Pre-diabetes)<br>40 Diabetes with HbA1c <7.0<br>30 Diabetes with HbA1c 7.0 – 7.9<br>20 Diabetes with HbA1c 8.0 – 8.9<br>10 Diabetes with Hb A1c 9.0 – 9.9<br>0 Diabetes with HbA1c ≥10.0
	Blood pressure	Appropriately measured systolic and diastolic blood pressure	**Metric:** Systolic and diastolic blood pressure (mm Hg)<br><br>**Scoring:**<br><u>Points Level</u><br>100 <120/<80 (Optimal)<br>75 120-129/<80 (Elevated)

			50 130-139 or 80-89 (Stage I HTN)<br>25 140-159 or 90-99<br>0 ≥160 or ≥100<br><br>Subtract 20 points if treated level

Supplementary Material

Table S2. Healthy Eating Index-2015 Components & Scoring Standards.

Component	Maximum points[1]	The standard for maximum score	The standard for a minimum score of zero
Adequacy			
Total Fruits[2]	5	≥0.8 cup equiv. per 1,000 kcal	No Fruit
Whole Fruits[3]	5	≥0.4 cup equiv. per 1,000 kcal	No Whole Fruit
Total Vegetables[4]	5	≥1.1 cup equiv. per 1,000 kcal	No Vegetables
Greens and Beans[4]	5	≥0.2 cup equiv. per 1,000 kcal	No Dark Green Vegetables or Legumes
Whole Grains	10	≥1.5 oz equiv. per 1,000 kcal	No Whole Grains
Dairy[5]	10	≥1.3 cup equiv. per 1,000 kcal	No Dairy
Total Protein Foods[6]	5	≥2.5 oz equiv. per 1,000 kcal	No Protein Foods
Seafood and Plant Proteins[6,7]	5	≥0.8 oz equiv. per 1,000 kcal	No Seafood or Plant Proteins
Fatty Acids[8]	10	(PUFAs + MUFAs)/SFAs ≥2.5	(PUFAs + MUFAs)/SFAs ≤1.2
Moderation			
Refined Grains	10	≤1.8 oz equiv. per 1,000 kcal	≥4.3 oz equiv. per 1,000 kcal
Sodium	10	≤1.1 gram per 1,000 kcal	≥2.0 grams per 1,000 kcal
Added Sugars	10	≤6.5% of energy	≥26% of energy
Saturated Fats	10	≤8% of energy	≥16% of energy

(1) Intakes between the minimum and maximum standards are scored proportionately.

(2) Includes 100% fruit juice.

(3) Includes all forms except juice.

(4) Includes legumes (beans and peas).

(5) Includes all milk products, such as fluid milk, yogurt, cheese, and fortified soy beverages.

(6) Includes legumes (beans and peas).

(7) Includes seafood, nuts, seeds, soy products (other than beverages), and legumes (beans and peas).

(8) Ratio of poly- and monounsaturated fatty acids (PUFAs and MUFAs) to saturated fatty acids (SFAs).

Adequacy components represent the food groups, subgroups, and dietary elements that are encouraged. Higher scores reflect higher intakes for these components because higher intakes are desirable.

Moderation components represent the food groups and dietary elements for which there are recommended limits to consumption. For moderation components, higher scores reflect lower intakes, because lower intakes are more desirable.

Supplementary Material

Table S3. Description of covariates.

Covariates	Description in NHANES
Age	Divided into three groups: 20-40 years old, >40 years old
Gender	Male and Female
Race	Mexican American, Non-Hispanic Black, Non-Hispanic White, Other Race
Educational level	Below high school, High School or above
Marital status	Yes: Married/Living with partner
PIR	Poor: <1.3; Not Poor:>=1.3
Smoking	Smoking status was grouped into never smoker (defined as <100 cigarettes in a lifetime), current smoker (defined as ≥100 cigarettes in a lifetime), and former smoker (defined as ≥100 cigarettes and had quit smoking)
Drinking	heavy drinking (≥4 drinks/day for men, ≥3 drinks/day for women, or ≥5 days of drinking in a month),<br>moderate drinking (≥3 drinks/day for men, ≥2 drinks/day for women, or ≥2 days of drinking in a month),<br>mild drinking (≤2 drinks/day for men, ≤1 drink/day for women, and ≥12 drinks in a year),<br>and never-drinking (total number of drinks in a year <12, and dietary alcohol content of 0%)
Physical activity	Active physical activity was defined as >599 MET, or >149 min of moderate physical activity, or >74 min of vigorous physical activity
Diabetes	Diabetes was defined as a history of previous diabetes, HbA1c level ≥6.5%, or fasting blood glucose level ≥126 mg/dL
Hypertension	The diagnostic criteria consist of self-reported hypertension history, the utilization of antihypertensive medication, a systolic blood pressure (SBP) ≥ 140mmHg, or a diastolic blood pressure (DBP) ≥ 90mmHg
Hyperlipidemia	(1) Triglyceride (TG) levels ≥ 150 mg/dl (1.7 mmol/L);(2) Total cholesterol (TC) levels ≥200 mg/dl (5.18 mmol/L);(3) Low-density lipoprotein (LDL) levels ≥ 130 mg/dl (3.37 mmol/L);(4)

	lowering drugs are also considered hyperlipidemia.

PIR, Ratio of family income to poverty.
